# Epidemiology, injury characteristics and clinical outcomes of bicycle and motorcycle accidents in the under 20 population: South Korea

**DOI:** 10.1186/s12873-022-00614-8

**Published:** 2022-03-31

**Authors:** Hyeokmin Yun, Sung Jin Bae, Jung Il Lee, Duk Hee Lee

**Affiliations:** 1 Department of Emergency Medicine, College of Medicine, Ewha Womans University, 1071 Anyangcheon-ro, Yangcheon-gu, Seoul, South Korea; 2 Department of Emergency Medicine, Chung-Ang University Gwangmyeong Hospital, College of Medicine, Chung-Ang University, Seoul, Republic of Korea; 3 Department of Orthopedic Surgery, Korea University Guro Hospital, 148, Gurodong-ro, Guro-gu, Seoul, South Korea

**Keywords:** Adolescence, Bicycle Injury, Fracture, Injury, Motorcycle Injury, Paediatric

## Abstract

**Background:**

Bicycles and motorcycles are a main means of transportation and leisure for individuals aged under 20 years in South Korea. We aimed to identify the epidemiology of injuries and describe and compare patterns of injury and clinical outcomes of two-wheel vehicle-related accidents in these individuals.

**Methods:**

We analysed data obtained from the National Emergency Department Information System for 401 emergency departments (EDs) from January 2016 to December 2018. We included patients aged < 20 years who experienced injuries while driving or a passenger on two-wheeled vehicles. We analysed patients with a bicycle-related injury and those with a motorcycle-related injury, and then compared two groups and performed a regression analysis for factors predicting severe trauma.

**Results:**

This study enrolled 54,342 two-wheel vehicle injury patients (37,410 bicycle and 16,932 motorcycle-related), of which, 86.8% (bicycle) and 94.9% (motorcycle) were males. External injuries were the most common. ED mortality was 9 (0.0%) for bicycles and 53 (0.3%) for motorcycles.

Overall, 3,346 (8.9%) patients were hospitalised with bicycle injuries and 4,096 (24.2%) with motorcycle injuries. Among admitted patients with bicycle-related injuries, 48.7% had upper extremity injuries and among those admitted patients with motorcycle-related injuries, 76.0% had lower extremity injuries.

Among hospitalised patients, the mean injury severity score (ISS) was 12.0 ± 12.6 in bicycle-related injury and 17.6 ± 15.4 in motorcycle-related injury. The number of patients with ISS ≥ 16 was 27.6% for bicycle related injuries and 45.2% for motorcycle-related injuries. The mean length of hospital stay was 191.5.8 ± 224.2 h for bicycle injury, and 359.6 ± 416.7 h for motorcycles. Hospital mortality cases were 0.2% with bicycle injury and 1.2% with motorcycle injury. Motorcycle-related injuries had more severe injury (ISS ≥ 16), with an adjusted odds ratio of 2.825 (95% confidence interval 2.610–3.059) compared to bicycle-related injuries.

**Conclusions:**

In the population aged under 20 years, two-wheel vehicle-related occurred predominantly in males. When using two-wheeled vehicles, motorcycle injuries were higher in patients aged over 14 years and were associated with higher ISS (≥ 16). Political efforts should be made to educate under 20 years of age for safe driving and to wear protective gear, including helmets to prevent severe injury.

## Background

Bicycles and motorcycles are the main means of transportation and leisure for individuals aged under 20 years in South Korea. However, the use of two-wheeled vehicles often leads to injuries. Injury is a leading cause of death and morbidity in adolescents aged 20 or younger [1]. Traffic accidents are one of the most common causes of emergency departments (ED) admission [2]. Bicycle and motorcycle crashes show different characteristics from other types of accidents, such as accidents in cars. Unlike car accidents, the body is directly exposed to the external environment in two-wheeler accidents. Because bicycles and motorcycles use only two wheels for balance, they can easily fall. In addition, they have a high probability of accidents owing to the influence of road surface and environment. As a result, fatal damage, such as head and limb injuries, may occur [3–5].

In South Korea, the adoption of cycling has steadily risen owing to increased interest in leisure activities and the government’s policy to encourage the use of bicycles by developing bicycle sharing systems and bicycle lanes [6]. The overall use of motorcycles is also gradually increasing owing to the demand for food-delivery services. As the use of bicycles and motorcycles increases, the number of related traffic accidents also increases. From 2010 to 2019, the number of traffic accidents involving motorcycles and bicycles increased from 17,672 to 20,898 and from 11,439 to 13,693, respectively [7].

Patients who visit the ED owing to motorcycle- and bicycle-related traffic accidents have varying severity of injuries and may also require hospitalisation. Previous studies have examined two-wheeled vehicle-related injuries [8–10]. Bicycles and motorcycles are easier to use for paediatric and adolescent age groups than other transportation. In South Korea, driving licenses for cars are permitted for individuals from age 18 years, whereas driving licenses for motorcycles are permitted for individuals from age 16 years. In addition, according to the World Health Organization, road traffic injuries, including motorcycle and bicycle accidents, are consistently one of the top three causes of death among young people [11]. Traffic accidents are the second leading cause of death in South Korea, and the third leading cause of death in children aged under 10 years [12].

Previous studies comparing bicycle and motorcycle-related accidents have shown differences in injury characteristics and outcomes [10, 13, 14]. Paediatric and adolescent injuries are an important public health concern because of their high global impact on death and disability [15].

This study aimed to identify the general epidemiology of two-wheeled vehicle-related injuries and determine injury patterns and clinical outcomes of motorcycle- and bicycle-related accidents in individuals under 20 years.

## Methods

### Setting and data collection

This study used prospectively collected data from the National Emergency Department Information System (NEDIS) between January 2016 and December 2018. The NEDIS started in 2003, and the number of participating emergency medical institutions has since increased. There are 36 regional emergency medical centres (Level 1), 117 local emergency medical centres (Level 2), and 119 local emergency medical rooms (Level 3) in South Korea. From 2016 to 2018, 399 (99.5%) emergency medical institutions participated in NEDIS data collection [16]. The information from patients who visited EDs was sent from each ED to the National Emergency Medical Centre database in real time.

In this study, all patients aged under 20 years with traffic accident injuries who visited EDs were identified. Patients who died at the scene of the accident and did not visit the ED could not be included. We included patients who were drivers or passengers of two-wheeled vehicles, and excluded any pedestrians involved in these accidents.

### Variables and outcome measures

The NEDIS collects demographic and clinical data: age, sex, ED visit date, ED visit time, geographic location of EDs, insurance types, helmet use, means of visit, consciousness of patients on arrival to the ED, systolic blood pressure, diastolic blood pressure, pulse rate, respiratory rate, diagnosis, injury severity score (ISS), and dispositions after ED care (discharge, transfer to another hospital, admission to general ward [GW], or intensive care unit [ICU]). In the case of admitted patients, data on final diagnosis and medical results on discharge were collected. We divided ED visit dates into spring (March to May), summer (June to August), autumn (September to November), and winter (December to February). The accident time was divided into dawn (00:00–05:59), morning (06:00–11:59), afternoon (12:00–17:59), and night (18:00–23:59).

We analysed the final diagnosis to categorise injuries (injury regions and fracture sites). Injury characteristics included the epidemiology and severity of injuries according to the Abbreviated Injury Scale (AIS) score and Injury Severity Score (ISS) [17]. ED disposition, type of discharge on admission, duration of hospitalisation, and mortality were analysed as clinical outcomes.

### Statistical analysis

We analysed patients with bicycle-related injuries and those with motorcycle-related injuries, separately. We compared and analysed variables of patients and injury-related characteristics between bicycle and motorcycle injuries. Categorical variables were analysed using the chi-square test. Student's t-test was used for continuous variables. Sex, age, area (urban or rural), and injury mechanism showed significant differences between ISS ≤ 15 and ISS ≥ 16 in the univariate analysis. To investigate factors predicting severe trauma (ISS ≥ 16), multivariable logistic regression analysis was performed using these factors. Statistical significance was set at a two-tailed *p*-value of < 0.05, and 95% confidence intervals were considered statistically significant. We used the Statistical Package for the Social Sciences Statistics for Windows version 21 (International Business Machines Corporation, Armonk, NY, USA).

## Results

**Fig. 1:**
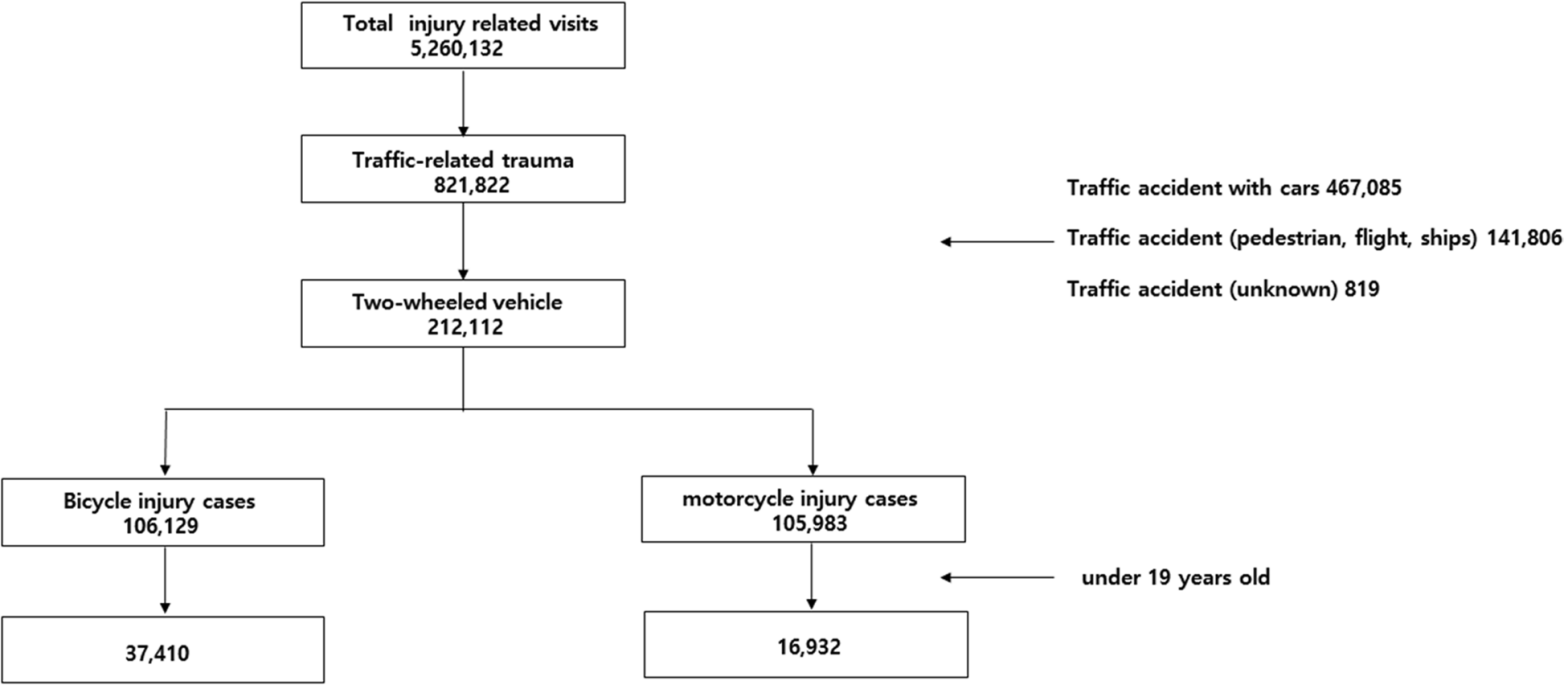
Flow gram

From January 2016 to December 2018, the total number of two-wheel vehicle-related injuries under 20 population in the nationwide EDs included 37,410 bicycle-related cases and 16,932 motorcycle-related cases. (Fig. 1).

**Fig. 2:**
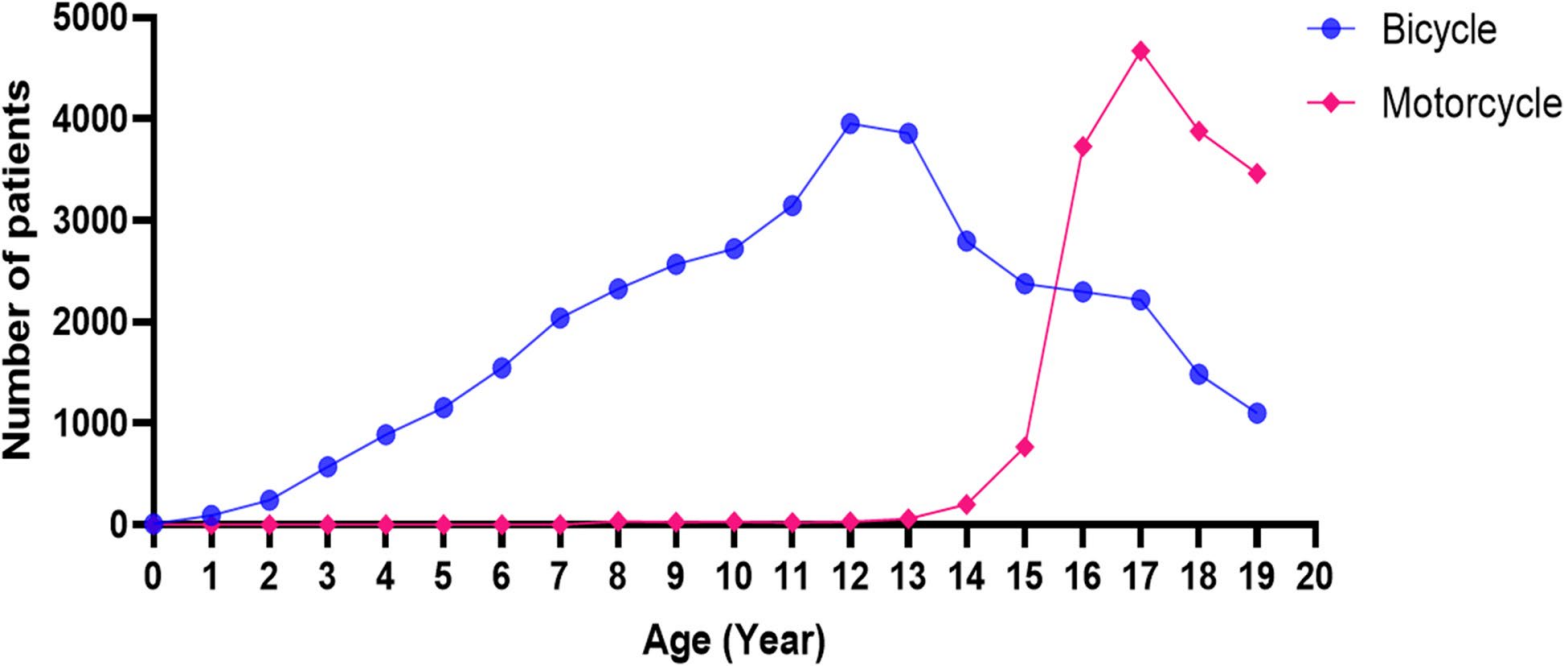
Incidence of two-wheeled vehicle injury by age

In the population aged under 20 years, bicycle patients were distributed among all ages, with peaks at 12–13 years. Motorcycle patients were mainly aged over 14 years, peaking at the age of 17 years. Motorcycle users increased geometrically above the age of 16 years because the license for motorcycles in South Korea is permitted from the age of 16 years (Fig. 2).

### ***Demographic characteristics of patients who visited EDs with two-wheel vehicle-related injury aged under 20 years ***(Table 1)

**Table 1:** Demographic characteristics of patients who visited the emergency department with two-wheeled vehicle-related injury

Variable	Bicycle		Motorcycle		Total	*p-value*
Number of injury cases	37,410	68.8%	16,932	31.2%	54,342	
Age	11.6 ± 4.0		17.1 ± 1.9		13.3 ± 4.3	0.000
Sex, male	32,478	86.8%	16,076	94.9%	48,554	0.000
**Season of injury**						0.000
Spring	11,373	30.4%	4,099	24.2%	15,472	
Summer	13,585	36.3%	4,793	28.3%	18,378	
Autumn	9,997	26.7%	4,724	27.9%	14,721	
Winter	2,455	6.6%	3,316	19.6%	5,771	
**Time of injury**						0.000
Morning (06:00 ~ 11:59)	1,379	3.7%	3,534	20.9%	4,913	
Afternoon (12:00 ~ 17:59)	4,279	11.4%	1,435	8.5%	5,714	
Night (18:00 ~ 23:59)	13,883	37.1%	3,484	20.6%	17,367	
Dawn (00:00 ~ 05:59)	17,869	47.8%	8,479	50.1%	26,348	
**Helmet use**						0.000
Yes	3,856	10.3%	7,903	46.7%	11,759	
No	33,554	89.7%	9,029	53.3%	42,583	
**Insurance type**						0.0001
National health care	26,893	71.9%	4,120	24.3%	31,013	
Traffic accident insurance	9,202	24.6%	11,792	69.6%	20,994	
Industrial accident insurance	2	0.0%	70	0.4%	72	
Medicaid	883	2.4%	441	2.6%	1,324	
Others	430	1.1%	509	3.0%	939	
**Means of visit**						0.0001
911	9,371	25.1%	10,499	62.0%	19,870	
hospital based ambulance	358	1.0%	595	3.5%	953	
Self-visit	27,456	73.4%	5,730	33.8%	33,186	
Others	255	0.7%	108	0.6%	363	
**Area**						0.0001
Urban area	27,561	73.7%	11,825	69.8%	39,386	
*number of urban injuries /100,000 population*	133.2		57.2			
Rural area	9,849	26.3%	5,107	30.2%	14,956	
*number of rural injuries /100,000 population*	111.2		57.6			
**Conciousness**						0.0001
Alert	37,254	99.6%	16,394	96.8%	53,648	
Altered	156	0.4%	538	3.2%	694	
**Systolic blood pressure (mmHg)**	103.4 ± 16.8		126..4 ± 27.6		124.6 ± 43.9	0.0001
**Diastolic blood pressure (mmHg)**	68.8 ± 11.0		75.5 ± 19.4		74.9 ± 30.1	0.109
**Pulse rate (beats/min)**	86.0 ± 25.8		86.2 ± 19.8		84.4 ± 20.8	0.0001
**Respiration rate (/min)**	19.3 ± 6.6		19.2 ± 8.4		19.4 ± 6.0	0.606
**Body temperature (℃)**	36.1 ± 2.6		36.4 ± 3.4		36.4 ± 2.8	0.0001

The mean ages were 11.6 ± 4.0 and 17.1 ± 1.9 years for patients involved in bicycle and motorcycle injuries, respectively. The rate of helmet use was 10.3% among patients with bicycle injuries and 46.7% among those with motorcycle injuries. Among patients with bicycle-related injuries, 71.9% were treated with National Health Care Insurance, and among those with motorcycle-related injuries, 69.6% were treated with traffic accident insurance. Among the patients, 25.1% of patients with bicycle injuries visited the ED via 911, and 62.0% of patients with motorcycle injuries visited the ED via 911. Both bicycle and motorcycle accidents occurred more frequently in urban areas than in rural areas. Approximately 0.4% of bicycle injuries and 3.2% of motorcycle injuries were altered consciousness.

### ***Comparison of injury patterns and clinical outcomes of patients aged under 20 years who visited the EDs with two-wheel vehicle-related injury ***(Table 2, 3)

**Table 2:** Injury patterns and clinical outcomes of patients who visited the emergency department with two-wheeled vehicle-related injury

**Variable**		**Bicycle**		**Motorcycle**		**Total**		***p-value***
		N	%	N	%	N	%	
**Number of patients in ED**		37,410		16,932		54,342		
**Region of injury**	Head and neck	6,316	16.9%	4,729	27.9%	11,045	20.3%	
	Face	2,492	6.7%	1,826	10.8%	4,318	7.9%	
	Chest	1,189	3.2%	1,146	6.8%	2,335	4.3%	
	Abdomen and pelvic contents	272	0.7%	307	1.8%	579	1.1%	
	External	21,144	56.5%	10,257	60.6%	31,401	57.8%	
	Extremities and pelvic girdle	54	0.1%	124	0.7%	178	0.3%	
	Upper extremities	12,127	32.4%	5,527	32.6%	17,654	32.5%	
	Lower extremities	11,161	29.8%	9,627	56.9%	20,788	38.3%	
	Spine	449	1.2%	747	4.4%	1,196	2.2%	
	Unspecified	15	0.0%	25	0.1%	40	0.1%	
**Number of fractures**	Total fractures	11,629		7,792		19,421		
**Fracture site**	Skull	418	3.6%	474	6.1%	892	4.6%	
	Facial	1,909	16.4%	1,631	20.9%	3,540	18.2%	
	Spine	96	0.8%	207	2.7%	303	1.6%	
	Rib, sternum	120	1.0%	176	2.3%	296	1.5%	
	Scapula	90	0.8%	75	1.0%	165	0.8%	
	Clavicle	939	8.1%	379	4.9%	1,318	6.8%	
	Humerus	660	5.7%	166	2.1%	826	4.3%	
	Forearm	5,953	51.2%	2,886	37.0%	8,839	45.5%	
	Hand	736	6.3%	416	5.3%	1,152	5.9%	
	Pelvic ring	44	0.4%	91	1.2%	135	0.7%	
	Acetabulum	4	0.0%	30	0.4%	34	0.2%	
	Femur	238	2.0%	477	6.1%	715	3.7%	
	Patella	74	0.6%	97	1.2%	171	0.9%	
	Tibia/fibula	85	0.7%	237	3.0%	322	1.7%	
	Foot	263	2.3%	450	5.8%	713	3.7%	
**ISS (mean ± SD)**		5.0 ± 6.1		9.0 ± 10.4		6.2 ± 7.9		0.000
**ISS ≥ 16**		2,172	5.8%	3,078	18%	5,250	9.70%	0.000
**ED disposition**	Discharge	33,765	90.3%	12,156	71.8%	45,921	84.5%	
	Transfer	258	0.7%	574	3.4%	832	1.5%	
	ICU admission	3,040	8.1%	3,388	20.0%	6,428	11.8%	
	GW admission	306	0.8%	708	4.2%	1,014	1.9%	
	Death	9	0.0%	53	0.3%	62	0.1%	
	Others	27	0.1%	50	0.3%	77	0.1%	

**Table 3:** Factors predicting severe trauma (ISS ≥ 16) in bicycle- and motorcycle-related injuries

Outcome: ISS ≥ 16	Univariate analysis			Multivariate logistic analysis		
	**OR**	**95% CI**	***p-value***	**Adjusted OR**	**95% CI**	***p-value***
Sex (male)	1.571	1.410–1.749	0.000	0.934	0.832–1.047	0.241
Age	1.242	1.168–1.321	0.000	1.055	1.044–1.066	**0.000**
Area (Rural)	1.078	1.021–1.140	0.000	1.174	1.100–1.253	**0.000**
Injury mechanism (Motorcycle)	3.604	3.400–3.821	0.000	2.825	2.610–3.059	**0.000**
Brain trauma	5.476	5.131–5.845	0.000	5.861	5.473–6.275	**0.000**

Bicycle- and motorcycle-related injuries were categorised according to the AIS regions. Among bicycle-related injuries,16.9% had head and neck injuries, 6.7% had facial injuries, 3.2% had chest injuries, 0.7% had abdominal and pelvic injuries, 56.5% had external injuries, 32.4% had upper extremity injuries, 29.8% had lower extremity injuries, and 1.2% had spinal injuries.

Among motorcycle-related injuries, 27.9% had head and neck injuries, 10.8% had facial injuries, 6.8% had chest injuries, 1.8% had abdominal and pelvic injuries, 60.6% had external injuries, 32.6% had upper extremity injuries, 56.9% had lower extremity injuries, and 4.4% had spine injuries.

Motorcycle injuries had a higher proportion of skull (6.1%), facial (20.9%), spinal (2.7%), and rib and sternum (2.3%) fractures than bicycle injuries (3.6%. 16.4%, 0.8%, and 1.0%, respectively).

Bicycle-related injuries included fractures of the clavicle (8.1%), humerus (5.7%), forearm (51.2%), hand (6.3%), pelvic ring (0.4%), femur (2.0%), patella (0.6%), tibia and/or fibula (0.7%), and foot (2.3%). Motorcycle-related injuries included fractures in the clavicle (4.9%), humerus (2.1%), forearm (37.0%), hand (5.3%), pelvic ring (1.2%), acetabulum (0.4%), femur (6.1%), patella (1.2%), tibia and/or fibula (3.0%), and foot (5.0%).

The mean ISS was 5.0 ± 6.1 for bicycle injuries and 9.0 ± 10.4 for motorcycle injuries (*P* < 0.000). In total, 2,172 (5.8%) patients with bicycle injuries and 30,278 (18.0%) with motorcycle injuries had ISS ≥ 16 (*P* < 0.000).

Disposition of ED in bicycle injuries was discharge, transfer, ICU admission, GW admission, and death in 90.3%, 0.7%, 8.1%, 0.8%, and 0.0% patients, respectively. Disposition of the ED in motorcycle injuries was discharge, transfer, ICU admission, GW admission, and death in 71.8%, 3.4%, 20.0%, 4.2%, and 0.3%, respectively.

The multivariable logistic regression analysis showed that sex was not a significant factor in severe trauma. Higher age and rural areas were statistically significant, with the odds ratios (ORs) of 1.055 (95% confidence interval [CI] 1.044–1.066) and 1.174 (95% CI 1.100–1.253), respectively. Motorcycle-related injuries had more severe injury (ISS ≥ 16), with an adjusted odds ratio (OR) of 2.825 (95% confidence interval [CI] 2.610–3.059) compared to bicycle-related injuries. Brain trauma was associated with severe trauma with an OR 5.861 (95% CI 5.473–6.275). These findings indicate that motorcycle injuries were associated with a higher number of severe injuries that required hospitalisation compared with bicycle injuries.

### ***Injury patterns and clinical outcomes of patients who were hospitalised with two-wheeled vehicle-related injury ***(Table 4)

**Table 4:** Injury patterns and clinical outcomes of patients who were hospitalised after two-wheeled vehicle related injury

**Variable**		**Bicycle**		**Motorcycle**		**Total**		***p-value***
**Number of Hospitalization patients**		3,346		4,096		7,442		
**Region of injury**	Head and neck	1,486	44.4%	2,430	59.3%	3,916	52.6%	0.000
	Face	467	14.0%	1,077	26.3%	1,544	20.7%	
	Chest	218	6.5%	587	14.3%	805	10.8%	
	Abdomen and pelvic contents	209	6.2%	342	8.3%	551	7.4%	
	External	1,270	38.0%	1,857	45.3%	3,127	42.0%	
	Extremities and pelvic girdle	28	0.8%	131	3.2%	159	2.1%	
	Upper extremities	1,631	48.7%	1,520	37.1%	3,151	42.3%	
	Lower extremities	1,057	31.6%	3,111	76.0%	4,168	56.0%	
	Spine	152	4.5%	461	11.3%	613	8.2%	
	Unspecified	6	0.2%	16	0.4%	22	0.3%	
**Number of Fractures**		2,564		4,469		7,033		0.000
**Fracture site**	Skull	280	10.9%	407	9.1%	687	9.8%	
	Facial	414	16.1%	989	22.1%	1,403	19.9%	
	Spine	57	2.2%	219	4.9%	276	3.9%	
	Rib, sternum	54	2.1%	139	3.1%	193	2.7%	
	Scapula	30	1.2%	52	1.2%	82	1.2%	
	Clavicle	279	10.9%	195	4.4%	474	6.7%	
	Humerus	344	13.4%	79	1.8%	423	6.0%	
	Forearm	652	25.4%	626	14.0%	1,278	18.2%	
	Hand	109	4.3%	250	5.6%	359	5.1%	
	Pelvic ring	23	0.9%	88	2.0%	111	1.6%	
	Acetabulum	3	0.1%	39	0.9%	42	0.6%	
	Femur	71	2.8%	454	10.2%	525	7.5%	
	Patella	12	0.5%	82	1.8%	94	1.3%	
	Tibia/fibula	163	6.4%	513	11.5%	676	9.6%	
	Foot	73	2.8%	337	7.5%	410	5.8%	
**ISS (mean ± SD)**		12.0 ± 12.6		17.6 ± 15.4		15.0 ± 14.5		0.000
**ISS ≥ 16**		922	27.6%	1,850	45.2%	2,772	37.2%	
**Clinical outcomes**	**Hospital LOS (hours)**	191.5.8 ± 224.2		359.6 ± 416.7		283.7 ± 353.4		0.000
	**In-hospital mortality**	6	0.2%	49	1.2%	55	0.7%	0.000

We analysed two-wheel vehicle related injury patients who were hospitalised in the ICU and GW. There were 3,346 bicycle injuries and 4,096 motorcycle injuries.

Hospitalised patients with bicycle injuries included 44.4% with head and neck injuries, 14.0% with face injuries, 6.5% with chest injuries, 6.2% with abdominal and pelvic injuries, 38.0% with external injuries 48.7% with upper extremity injuries, 31.6% with lower extremity injuries, and 4.5% with spinal injuries.

Hospitalised patients with motorcycle-related injuries included 59.3% with head and neck injuries, 26.3% with face injuries, 14.3%with chest injuries, 8.3%with abdominal and pelvic injuries, 45.3% with external injuries, 37.1% with upper extremity injuries, 76.0%with lower extremity injuries, 11.3%with spinal injuries.

Bicycle injuries included fractures of the skull (10.9%), face (16.1%), spine (2.2%), and sternum (2.1%). Motorcycle injuries included fractures of the skull (9.1%), face (22.1%), spine (4.9%), and sternum (3.1%).

A total of 2,564 patients with fractures were hospitalised after a bicycle accident. Bicycle-related injuries included fractures of the clavicle (10.9%), humerus (13.4%), forearm (25.4%), hand (4.3%), pelvic ring (0.9%), femur (2.8%), patella (0.5%), tibia and/or fibula (6.4%), and foot (2.8%). The 4,469 fractures in motorcycle-related accidents included fractures of the clavicle (4.4%), humerus (1.8%), forearm (14.0%), hand (5.6%), pelvic ring (2.0%), acetabulum (0.9%), femur (10.2%), patella (1.8%), tibia and/or fibula (11.5%), and foot (7.5%).

The mean ISS score for hospitalised patients was 12.0 ± 12.6 for bicycle injury and 17.6 ± 15.4 for motorcycle injury. ISS ≥ 16 was observed in 27.6% hospitalised patients after bicycle accidents and (45.2% hospitalised patients after motorcycle accidents. The mean length of hospital stay (LOS) was 191.5.8 ± 224.2 h for bicycle injury, while the mean LOS was 359.6 ± 416.7 h for motorcycle injury. There were 0.2% cases of hospital mortality with bicycle injuries and 1.2% with motorcycle injuries.

## Discussion

This is the first nationwide study to analyse the characteristics of two-wheel vehicle injuries and clinical outcomes in individuals younger than 20 years in South Korea. This population-based study using NEDIS data evaluated the difference between bicycle- and motorcycle-related injuries presenting to EDs in South Korea between 2016 and 2018. This study reported the incidence of injury and fracture sites as well as the demographic characteristics of patients, and categorised the injury region according to the AIS score and calculated ISS for all adolescent patients who visited the ED. We separately analysed hospitalised patients’ injury sites, fracture sites, ISS, and hospital length of stay. Previous studies have mainly focused on individual studies of bicycle or motorcycle crashes or comparative studies targeting the elderly [8, 18]; however, this study compared bicycle and motorcycle injuries among adolescents.

The Road Safety Report showed road deaths according to age groups in 2018 and revealed that the number of road deaths decreased by 28% among 0–20-year-old individuals after road safety improvements. Adolescents have a relatively low mortality rate on Korean roads compared with other international road traffic and accident databases. Mortality rates ranged from 0.6 for in individuals aged 0–14 years to 3.7 in those aged 21–24 years. This may be because young people in Korea tend to start driving late [19, 20]. In Korea, individuals older than 18 years can obtain a driver’s license for cars; however, a motorcycle license is available from the age of 16 years. In this study, it can be seen that the rate of bicycle accidents and motorcycle accidents decreased and increased, respectively, when patients were aged 14 years.

South Korea has four seasons, and this study shows that accidents occur frequently from spring to autumn and decrease in winter. Nurmi et al. reported that paediatric bicycle- and motorcycle-related accidents occur during more favourable weather conditions [21]. This may be because of the reduced use of two-wheel vehicles owing weather-related hinderances in winter compared to other seasons. Beck et al. reported that crashes commonly occur during daylight hours and in clear weather conditions [22]. This study showed that almost half of the injuries (bicycle, 47.8%; motorcycle, 50.1%) occurred in the dark, whereas the rate of injuries was the lowest in daylight time. This characteristic may be due to the low usage of vehicles because the group agedunder 20 years is in schools or nurseries during that time. The lack of light at night may make it difficult for other cars to notice two-wheeled vehicles or drivers of two-wheeled vehicles to see the road surface or structures clearly [23]. It seems that it is necessary to provide more education on protective equipment, etc. for driving in the dark to individuals aged under 20 years.

Although there have been many previous studies that have demonstrated the protective effects of helmets [24, 25], this study showed that only 10% of bicycle-injured patients and less than 50% of motorcycle-injured patients used helmets. A previous study on bicycle-related injury in the ED in South Korea during 2012–2014 showed that the use of helmets was nearly 20% [18] but decreased in 2016–2018 during the current study period. The helmet use rate of motorcycle riders was 85% in 2018, as surveyed by the Korea Road Traffic Authority (KoROAD). There was a large difference between KoROAD and this study in terms of the rate of individuals wearing helmets. There are two possible reasons for this finding. First, KoROAD’s measurements were based on the roads around traffic accident cases or on specific samples. Second, this study may not reflect the overall helmet usage rate in the population, as we only considered the helmet usage rate of patients using two-wheeled vehicles who visited the ED after injury [19]. If the actual motorcycle rider's helmet wearing rate was 85%, the helmet usage rate of the injury group visiting the ED was very low. Canzi analysed and reported the effect of injury on different body parts on mortality in road motorcycle accidents admitted to a level I trauma centre [26]. In this study, in those younger than 20 years, 9 deaths due to bicycle accidents and 53 due to motorcycle accidents were reported. Approximately 33.3% of bicycle deaths and 26.4% of motorcycle deaths were caused by head trauma. The effect of brain trauma on ISS ≥ 16 had an OR of 5.861. Two-wheeler vehicle users should recognise the importance of wearing a helmet, and a policy effort to encourage wearing a helmet is needed. This study showed that 86.8% of bicycle-related injuries and 94.9% of motorcycle-related injuries occurred in men. Previous studies have shown that men have a higher proportion of injuries than women [21, 27, 28].

In a report on orthopaedic characteristics of bicycle injuries in South Korea. Fractures of the forearm and shoulder were the most common orthopaedic injuries. This was a study of all age groups at a single institution [29]. This study showed that bicycle injuries occurred more frequently in the upper extremities than in the lower extremities (32.4% vs 29.8%), whereas motorcycle injuries occurred more frequently in the lower extremities than in the upper extremities (56.9% vs 32.6%) in the ED. An ISS ≥16 was more frequent in motorcycle injuries than in bicycle injuries, which means more severe injuries that need to be hospitalised in motorcycle injuries. We analysed two-wheeled vehicle-related injury patients who were admitted to the ICU and GW. These results indicate that the severity of motorcycle-related accidents is higher. 

Most childhood traffic accidents are mild and require only minor treatment [21]. In the present study, the mortality rate was low. However, motorcycle mortality was seven times higher than that for bicycle injuries. We investigated both the disposition and clinical outcomes at both points; at discharge from the ED, and discharge after hospitalisation. This is because it would have been difficult to determine the overall mortality rate and injury that required hospitalisation if only the ED was reported. The national data used in this study were transmitted upon evacuation from the ED or upon discharge after hospitalisation. The average ISS was higher for motorcycle-related injuries, and patients with ISS ≥16 were also more likely to have been riding a motorcycle. Indicating that even when the same region is injured, the severity of motorcycle-related accidents is higher. Patients with motorcycle accidents were more severely injured than those with bicycles, hospital LOS and mortality rates were also higher. Comparing the entire patient visiting the ED with the hospitalised patient, the frequency order of the injured area was different. Many of the hospitalised patients were injured in the head and neck, face, and lower extremities. These injuries require hospitalisation and affect the increase in ISS. Lower extremity injuries were higher in motorcycle accidents than in bicycle accidents. It could have a negative impact on the return to daily life and quality of life. Zibung et al. reported that more than 70% of bicycle trauma patients suffered physically for over six months after their crash, even though the trauma was mild. Cervical and facial injuries and ISS ≥15 are risk factors for impaired quality of life [30]. Patients experiencing motorcycle accidents are more vulnerable to lower extremity injuries than those experiencing bicycle accidents. Kohler et al. reported that trauma to the lower extremities led to physical distress and ongoing social and economic costs, while injuries affecting mobility have widespread levels of injuries and economic consequences for the patient and also affected the family [31]. One-year post-injury, patients with lower extremity injury reported limitations in walking (46%), inability to return to work (22%), depression (39%), and post-traumatic stress disorder (18%). Long-lasting physical and psychological burdens may impede recovery and alter the lifestyle of patients with lower extremity injuries [32]. In particular, major trauma in adolescents was associated with significant and marked deficits in quality of life throughout the 24-month follow-up period [33].

There are many concerns regarding the safety of two-wheeled vehicles. The challenges for improving the safety of powered two-wheelers relate to the user, other road users, and the road environment. In some countries, even for proven safety measures, such as helmets, it is difficult to obtain rider approval [34]. In South Korea, helmets have become compulsory for users of all motorised two-wheelers since 2007. A systematic review and meta-analysis of bicycle injury and helmet use showed that bicycle helmets reduced serious head, face, and fatal head injury [24]. However, the rate of wearing a helmet on motorcycles and bicycles was low. Policies should be changed to recommend safety measures. Policies should strengthen and monitor laws that state the use of motorcycle helmets for both drivers and passengers, and laws should be enacted to restrict children from riding together. It is also necessary to legislate the use of bicycle helmets. In addition, the use body protectors is recommended for two-wheel vehicle riders. Upper and lower extremity injuries are the most common injuries, so using arm, knee, and joint protectors may help to prevent extremity injuries [35]. In addition, since most patients visit the ED at night, wearing protective gear with reflective lighting may help to prevent two-wheel vehicle crashes [36, 37].

Our study had several limitations. In this study, information on weather and road types was not considered. These factors may have affected the severity of the injury. We also had no information on the patients’ medical history or other laboratory tests that could affect the patient's outcome. Information on several known and potential risk factors that could affect the severity, such as the speed at the time of the accident, the location of the accident, the weather, the license acquisition status, and engine displacement could not be obtained. This study analysed data from drivers and passengers. There is a possibility that the injury may be different between riders and passengers in the same vehicle at the same speed, so it would be helpful to study the safety of passengers by analysing only passengers in future studies. In addition, mopeds were not coded separately. According to the NEDIS guidelines, mopeds are classified as motorcycles. However, it is possible that several emergency departments misclassified moped as bicycles. Minor injuries could be treated at the outpatient clinic outside the ED and there might be cases that were fatal, thus precluding the need for an ED visit; therefore, this study was not representative of all two-wheel vehicle injuries. Finally, re-admission or post-outpatient hospitalisation after discharge from the ED was not monitored.

## Conclusions

In the population aged under 20 years, two-wheeled vehicle-related injuries had a very low mortality rate and occurred predominantly among males. Motorcycle injuries increased dramatically among individuals aged over 16 years, which is the required age for obtaining a license, and had a higher ISS (≥ 16), intensive care unit admission rate, and mortality rate than bicycle injuries. Injury severity was higher in motorcycle-related accidents (adjusted OR 2.787 [95% CI 2.582–3.009]) than in bicycle-related accidents. Bicycle accidents predominantly cause upper limb fractures, whereas motorcycle accidents predominantly cause lower limb fractures. Adolescent injuries may cause long-lasting physical and psychological problems. Preventive measures according to the mean and age groups are required. To reduce injuries, adequate education regarding accidents and the importance of wearing protective devices should be provided to the youth when they obtain a motorcycle license.

## Data Availability

The datasets generated during and analyzed during the current study are not publicly available due to the National Emergency Medical Center is the authority for accessing the data analyzed, and there are ethical restrictions on sharing a dataset because the data contain potentially identifying information. But datasets are available from the corresponding author on reasonable request.
